# Somatic comorbidity in children and adolescents with psychiatric disorders

**DOI:** 10.1007/s00787-019-01313-9

**Published:** 2019-03-20

**Authors:** Sara Agnafors, Anna Norman Kjellström, Jarl Torgerson, Marie Rusner

**Affiliations:** 1 Division of Children’s and Women’s Health, Department of Clinical and Experimental Medicine, Linköping University, 581 83 Linköping, Sweden; 2 Department of Research, Södra Älvsborgs Hospital, Borås, Sweden; 3 Department of Data Management and Analysis, Head Office, Region Västra Götaland, Skövde, Sweden; 4 Department of Psychosis, Sahlgrenska University Hospital, Göteborg, Sweden; 5 Institute of Health and Care Sciences, Sahlgrenska Academy, University of Gothenburg, Göteborg, Sweden

**Keywords:** Comorbidity, Mental health, Somatic health, Children

## Abstract

In the adult population, psychiatric disorders are associated with somatic illness. Explanatory life style factors have been found, but also a failure to recognize somatic illness in this group. Another factor is side effects from long-term use of antipsychotic drugs. Given the psychiatric-somatic comorbidity in the adult population, it is of interest to investigate whether an association exists already during childhood. The aim of the present study was to investigate the frequency of somatic illness in children and adolescents with a psychiatric diagnose. Data were obtained from the regional health care database Vega, Sweden. Psychiatric and somatic diagnoses obtained during 2011–2013 for individuals aged 3–18 years were extracted. Descriptive statistics were used to examine difference in somatic morbidity between children with and without psychiatric diagnoses. Logistic regression was used in age-stratified models to test the association between psychiatric and somatic diagnoses. Anxiety and behavioral disorders were associated with all somatic conditions investigated at nearly all ages. The same applied to substance use, investigated at age 9–18 years. Affective disorders were associated with all somatic conditions at age 12–18 years. Psychotic conditions were associated with asthma, bowel disorders and myalgia in adolescents. Children with psychiatric disorders are at remarkably high risk for concurrent somatic illness. The associations span across many types of conditions and across all ages. The results support the need for awareness of somatic morbidity in child and adolescent psychiatric clinical settings, and the need for coordinated health care for children with comorbid states.

**Electronic supplementary material:**

The online version of this article (10.1007/s00787-019-01313-9) contains supplementary material, which is available to authorized users.

## Background

Psychiatric disorders, such as depression and schizophrenia, are associated with increased somatic morbidity and mortality among adults [1]. Previous studies have shown increased mortality in ischemic heart disease [2] and shorter life span in adults diagnosed with severe mental illness [3, 4]. Several possible explanations for these disparities have been suggested. Stigmatization and cognitive difficulties might hinder the establishment of health care contacts, to follow treatment regimens and screening programs [5]. Organizational structures that complicate cooperation between somatic and psychiatric health care as well as lack of knowledge about psychiatric-somatic comorbidity are other hindering factors [6]. Among individuals with schizophrenia, risk factors such as smoking, substance abuse, lack of physical activity and unhealthy diets are more prevalent [7]. Moreover, prolonged use of antipsychotic drugs known to be associated with metabolic side effects [8], and economic disadvantage resulting in non-adherence to medical treatments and health care visits, increases the risk for somatic ill-health. Researchers and authorities have raised concern over somatic symptoms in this population being unrecognized and call for increased awareness to promote health care access and treatment.

Given the association between mental and somatic illness in the adult population, it is of interest to investigate whether a psychiatric-somatic comorbidity exists already during childhood and adolescence. The prevalence of severe mental illness such as psychosis is very low during childhood; however, adult individuals diagnosed with psychosis have often shown psychiatric symptoms or been diagnosed with other psychiatric disorders during childhood [9]. In general, mental health problems during childhood are associated with mental health problems in adulthood [10], and previous research have indicated a complex pattern of both homotypic and heterotypic continuity [11].

The literature on somatic ill-health in children and adolescents with psychiatric diagnoses is comparably sparse. Large-scale studies investigating associations between a broad spectrum of psychiatric and somatic clinically diagnosed disorders are lacking. However, a few population-based as well as clinical studies have shown increased somatic illness in children and adolescents with psychiatric disorders.[12–15]. Goodwin et al. found an association between mental illness during childhood and chronic somatic illnesses such as asthma, overweight and epilepsy in young adulthood [16]. In a recent study, ADHD was found to be associated with the development of obesity over time [17]. In a large population-based study, the cost for somatic health care was found to be almost two times as high for children with psychiatric diagnoses than in children without psychiatric diagnoses [18].

With the starting point in somatic disorders, asthma, atopic diseases and overweight has been associated with anxiety [19–21]. Similar findings have been noted for attention deficit hyperactivity disorder (ADHD) [22, 23]. Increased rates of depression have been found in children with diabetes [24]. Studies on comorbidity between mental and somatic disorders have almost exclusively focused on psychiatric symptoms as a result of somatic morbidity.

The aim of the present study was to investigate the frequency of somatic illness in children and adolescents with psychiatric diagnoses. The hypothesis is that mental illness during childhood and adolescence is associated with an increased frequency of somatic illness. The study adds to the existing literature by (1) investigating a broad range of somatic morbidity in children with psychiatric diagnoses and (2) the use of a large population-based sample, stratified by age from early childhood to late adolescence.

## Methods

### Subjects

The study population was based on individuals aged 3–18 years in 2012, who were residents of Västra Götaland in 2012. Data were extracted from the regional health care database Vega. Vega includes information about all primary and specialist health care in Region Västra Götaland, Sweden, and both public and private caregivers are obliged to deliver data. Vega contains information about date of contact, type of contact, healthcare provider, diagnoses, operations, health centers and hospitals and age and sex of the patient. Diagnoses included in Vega are coded according to the International Statistical Classification of Diseases and Related Health Problems 10th revision, ICD-10.

All individuals aged 3–18 years in 2012 with a somatic or psychiatric diagnose registered in 2011–2013 as defined in Table 1 were extracted from Vega and included in the study (*n* = 48,289). Based on gender and age, the study population was then completed with those individuals aged 3–18 years in region Västra Götaland in 2012 without these diagnoses, resulting in a population of 281,476 individuals. The latter information was retrieved from Statistics Sweden.

**Table 1:** Classification of psychiatric and somatic diagnoses

	ICD-10 code
Psychiatric diagnoses	
Mental and behavioral disorders due to psychoactive substance use	F10–F19
Schizophrenia, schizotypal and delusional disorders	F20–F29
Affective (mood) disorders	F30–F39
Neurotic, stress-related and somatoform disorders	F40–F48
Behavioral and emotional disorders with onset in childhood and adolescence	F90–F98
Somatic diagnoses	
Obesity	E66
Asthma	J45–J46
Diabetes type 1	E10
Irritable bowel syndrome and other functional intestinal disorders	K58, K59
Dermatitis and eczema	L20–L30
Myalgia	M79.1
Migraine	G43
Other headache syndromes	G44

*ICD *International Statistical Classification of Diseases and Related Health Problems 10th revision

Psychiatric diagnoses included were chosen to cover common childhood psychiatric conditions. Somatic diagnoses were selected in part based on previous studies [12, 13] and in part exploratory. Somatic illness diagnosed in 2011–2013 was compared between individuals with and without psychiatric diagnose in 2011–2013. Subjects with eating disorders (ICD-10 F50) or developmental delays (ICD-10 F70–F79, F82–F83, F84.2–F84.4, F84.8–F84.9, F88–F89) were excluded from the study population since these conditions could be primarily associated with somatic illness and, therefore, potentially bias the results. For detailed description on psychiatric and somatic diagnoses, see Table 1. To simplify for the reader, the term anxiety is used for neurotic, stress-related and somatoform disorders, and behavioral disorders is used for behavioral and emotional disorders with onset in childhood and adolescence. The term psychotic conditions is used for schizophrenia, schizotypal and delusional disorders.

### Data analysis

Descriptive statistics were used to illustrate frequencies of psychiatric and somatic diagnoses. Chi^2^ was used to investigate differences in somatic diagnoses between children diagnosed with psychiatric disorders, and those who were not. Logistic regression was used to examine the risk for somatic diagnoses in children with psychiatric diagnoses. The age-stratified models were controlled for gender, and the models that were not stratified by age were controlled for gender and age. Data were divided in five categories based on age in 2012: 3–5 years, 6–8 years, 9–11 years, 12–14 years and 15–18 years. When analyses resulted in cases of five or fewer, data were not shown to maintain the integrity of the study participants. Narrow age categories were chosen due to the physical and mental development during childhood. A *p *value < 0.001 (two-sided) was considered statistically significant in descriptive statistics. Results from logistic regressions are presented with corresponding odds ratios (OR) and 95% confidence intervals (CI). All analyses were conducted using SPSS version 24 (IBM Corporation, Armonk, NY, USA).

## Results

### Frequencies

Frequencies of psychiatric diagnose categories and somatic diagnoses are found in Table 2. Prevalence rates were somewhat lower than previously noted.

**Table 2:** Frequencies of psychiatric and somatic diagnoses based on age group during the study period

Age	3–5 *N* = 56,841	6–8 *N* = 53,379	9–11 *N* = 50,311	12–14 *N* = 48,589	15–18 *N* = 72,356	3–18 (tot) *N* = 281,476
Psychiatric conditions						
Anxiety	*N* = 89 0.2%	*N* = 355 0.7%	*N* = 928 1.8%	*N* = 1432 2.9%	*N* = 3560 4.9%	*N* = 6364 2.3%
Affective	*N* = 8 0.0%	*N* = 58 0.1%	*N* = 215 0.4%	*N* = 654 1.3%	*N* = 2273 3.1%	*N* = 3208 1.1%
Behavior	*N* = 990 1.7%	*N* = 2309 4.3%	*N* = 2141 4.3%	*N* = 1950 4%	*N* = 2339 3.2%	*N* = 9729 3.5%
Psychosis	X	X	X	*N* = 10 0%	*N* = 66 0.1%	*N* = 81 0%
Substance use	X	X	*N* = 10 0%	*N* = 123 0.3%	*N* = 831 1.1%	*N* = 970 0.3%
Somatic conditions						
Obesity	*N* = 267 0.5%	*N* = 627 1.2%	*N* = 888 1.8%	*N* = 818 1.7%	*N* = 793 1.1%	*N* = 3393 1.2%
Asthma	*N* = 3953 7%	*N* = 2804 5.3%	*N* = 2583 5.1%	*N* = 2577 5.3%	*N* = 2992 4.1%	*N* = 14,909 5.3%
Diabetes type 1	*N* = 89 0.2%	*N* = 144 0.3%	*N* = 218 0.4%	*N* = 310 0.6%	*N* = 530 0.7%	*N* = 1291 0.5%
Bowel	*N* = 3179 5.6%	*N* = 2330 4.4%	*N* = 1832 3.6%	*N* = 1192 2.5%	*N* = 1518 2.1%	*N* = 10,051 3.6%
Eczema	*N* = 4395 7.7%	*N* = 3397 6.4%	*N* = 2838 5.6%	*N* = 2274 4.7%	*N* = 3398 4.7%	*N* = 16,302 5.8%
Myalgia	*N* = 257 0.5%	*N* = 492 0.9%	*N* = 813 1.6%	*N* = 1062 2.2%	*N* = 2063 2.9%	*N* = 4687 1.7%
Migraine	*N* = 82 0.1%	*N* = 237 0.4%	*N* = 360 0.7%	*N* = 564 1.2%	*N* = 814 1.1%	*N* = 2057 0.7%
Headache	*N* = 33 0.1%	*N* = 143 0.3%	*N* = 280 0.6%	*N* = 426 0.9%	*N* = 929 1.3%	*N* = 1811 0.6%

X = 5 individuals or fewer

### Descriptives

In general, large difference in somatic illness was noted between children with anxiety conditions and those without (Supplementary material). At age 3–5 years significant differences were found for asthma, eczema and bowel symptoms. In the three oldest age groups and in the total population, significant differences between groups were found for all somatic conditions investigated. Notable differences were found in frequencies of obesity, where 4.5% (*n* = 64) of children aged 12–14 years with anxiety were diagnosed with obesity compared to 1.6% (*n* = 754) of children the same age without anxiety (Pearson *χ*^2^ 69.18, *p* < 0.001). Similar differences were found at age 15–18 years.

For affective disorders, few significant differences emerged up to age 8 years due to the low prevalence rates (Supplementary material). In the two oldest groups, adolescents with affective disorders were more likely to be diagnosed with all somatic conditions included, with the only exception of diabetes type 1 in age 12–14 years. Obesity, bowel syndromes, migraine and headache were more than four times as common in children with affective disorders age 15–18 years. For example, 4.9% (*n* = 111) of adolescents with an affective disorder were diagnosed with headache, compared to 1.2% (*n* = 818) of adolescents without an affective disorder (Pearson *χ*^2^ 239.89, *p* < 0.001). When no age stratification was used in analysis, significant differences were found for all somatic diagnoses included.

From age 6 years and above, and in the total population, most somatic disorders included were approximately twice as common in children and adolescents with behavioral disorders as in those without (Supplementary material). 18.8% (*n* = 186) of the children aged 3–5 years with behavioral disorders were diagnosed with asthma, compared to 6.7% of children without behavioral disorders (Pearson *χ*^2^ 218.03, *p* < 0.001). Obesity was more than five times more common in 15–18 year old’s with behavioral disorders (5.5%, *n* = 123) than in those without (1.0%, *n* = 670) (Pearson *χ*^2^ 39.186, *p* < 0.001).

Due to the low prevalence of psychotic conditions under the age of 12 years, only adolescents aged 12–18 years were included in the statistical analyses. For many diagnoses, cases were fewer than five and results are thus not reported. 9.2% (*n* = 7) of adolescents with psychosis were diagnosed with myalgia compared to 2.6% (*n* = 3118) of study participants without psychosis (Pearson *χ*^2^ 13.27, *p* < 0.001). Significant differences were also found for asthma and bowel symptoms (Supplementary material).

Since there were no diagnoses of substance use below the age of 9 years, only children and adolescents aged 9–18 years were included in the statistical analyses. Children and adolescents who had a diagnose of substance use were more prone to be diagnosed with all somatic conditions investigated (Supplementary material). For example, 4.1% (*n* = 40) were diagnosed with headache, compared to 0.9% (*n* = 1595) in the remaining study population (Pearson *χ*^2^, 104.63, *p* < 0.001). Corresponding numbers for obesity were 2.8% (*n* = 27) in the group diagnosed with substance use compared to 1.5% (*n* = 2472) in those with no substance use diagnose (Pearson *χ*^2^ 12.14, *p* < 0.001).

### Logistic regression

**Fig. 1:**
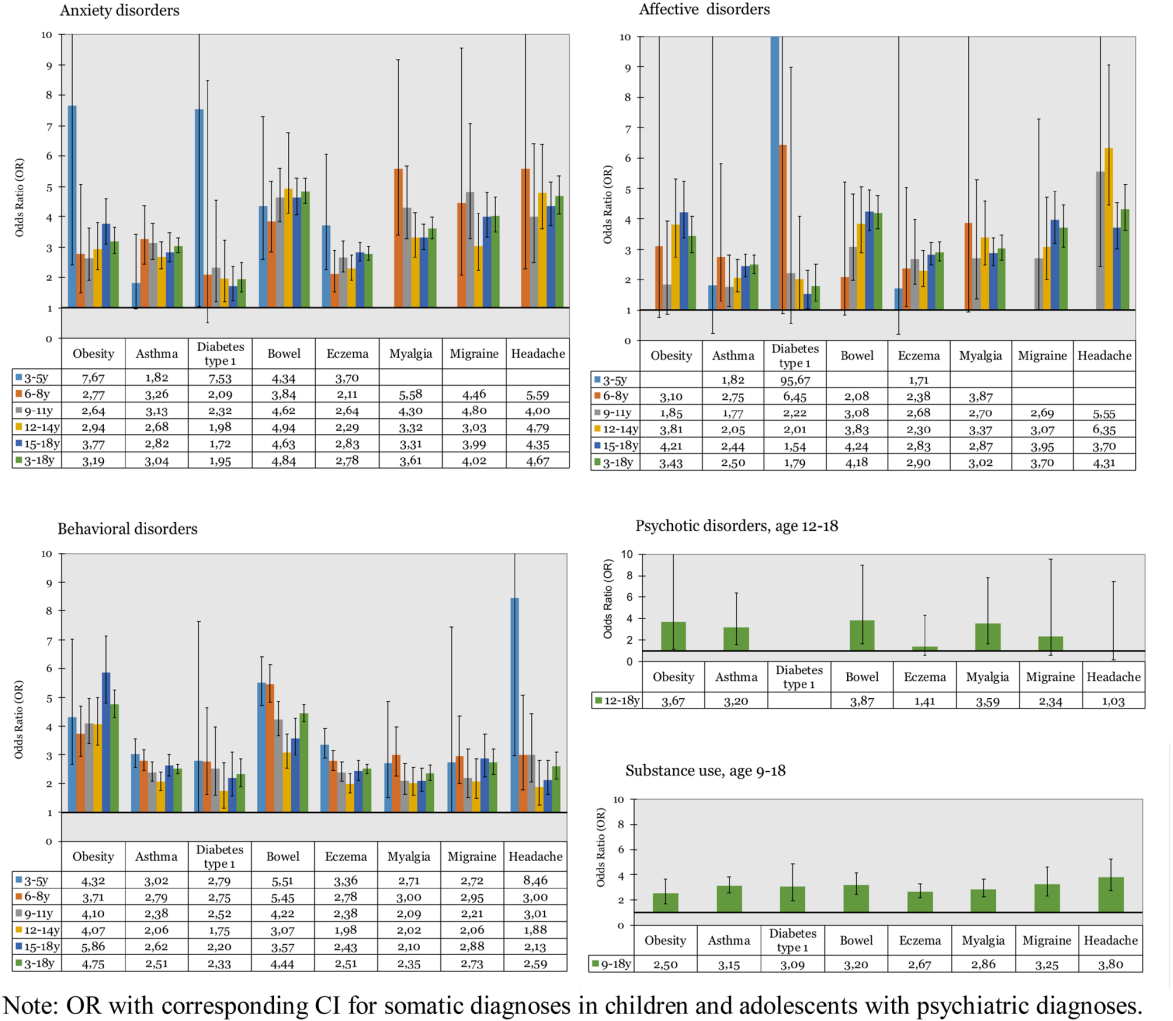
Odds ratios for somatic disorders in children and adolescents with psychiatric diagnoses

Results of the logistic regressions are presented in Fig. 1. Anxiety conditions were associated with all somatic conditions investigated, at nearly all ages. When analyses were conducted on the total population, age 3–18 years, significant associations were found for all somatic conditions. OR ranged from 1.72 (CI 1.26–2.36) for diabetes type 1 at age 15–18 years, to 7.67 (CI 2.41–24.41) for obesity at age 3–5 years.

Affective disorders were not associated with somatic conditions in the youngest children. OR increased with age, and from age 12 years, the associations were significant for almost all somatic conditions investigated (Fig. 1). With all age groups included, significant associations were noted for all somatic conditions. Extreme numbers were noted for diabetes type 1 (OR 95.67, CI 84.06–692.70) in age 3–5 years due to the low frequencies in this group.

Regarding behavioral disorders, significant associations were found for all somatic conditions investigated with the only exception of migraine in 3–5 year olds. In general, the associations were stronger for obesity where OR ranged from 3.7 at age 6–8 years, to 5.9 at age 15–18 years. When all ages were included, significant associations were found for all somatic diagnoses.

Significant associations were found between psychotic disorders and obesity, asthma, bowel symptoms and myalgia, with OR ranging from 3.2 to 3.9.

For substance use, significant associations were found for all somatic conditions included in the study. OR varied between above two for obesity (OR 2.50, CI 1.70–3.68) to just below four for headache (OR 3.80, CI 2.75–5.24).

## Discussion

The aim of the present study was to examine the frequency of somatic disorders in patients with psychiatric morbidity during a 3-year span in a large population-based cohort of 281,000 children and adolescents in western Sweden. In general, remarkably large and highly significant differences in prevalence rates of a broad range of somatic disorders and symptoms could be seen as early as preschool age, and these differences persisted during middle childhood and adolescence.

Obesity, which is an important risk factor for cardiovascular disease and premature mortality, was more prevalent in children and adolescents with anxiety, affective, and behavioral disorders. Remarkably high OR were noted for obesity, and the associations were found already at preschool age. Development of obesity has been noted previously in children with ADHD; however, the mechanisms are not known [17]. Considering the large effect sizes noted in the present study, and the long-term risk associated with obesity, the association between childhood psychiatric disorders and weight gain needs to be addressed in clinical practice.

Immunologic disorders such as asthma and eczema were approximately two to three times as common in children and adolescents with psychiatric disorders as in those without. The strength of the association did not vary with age and was consistent over the spectrum of psychiatric diagnoses. Atopic diseases have previously been found to be associated with anxiety, depression and ADHD [19, 20, 22]. Similarly, psychotic experience in adolescence has been linked to parental reports of atopy during childhood; however, effects were small [25]. Diabetes type 1 was found to be more common in children with anxiety, affective and behavioral disorders. Associations between diabetes type 1 and psychiatric symptoms have previously been noted and suggested to be impacted by poor glycemic control [26]. Longitudinal studies examining whether the somatic condition preceded the psychiatric or vice versa, would be of great interest to further understand this association.

The increased morbidity and mortality in adults with severe mental illness has been attributed to long-term use of antipsychotic medication, cognitive disability and associated risk behaviors [7]. The clear associations between mental and somatic ill-health already in early childhood cannot be attributed to these factors. The present study has a cross-sectional design and is thereby purely descriptive, however, longitudinal associations between psychiatric and somatic illness have been shown to be bidirectional [27]. In this study, comorbidity was present at all ages, and across all psychiatric diagnose groups. Thus, the findings call for further research into comorbidity and raises questions about possibly shared pathophysiology as well as epigenetic and immunological pathways. A couple of possible explanations will be discussed below.

First, children with access to psychiatric care have repeated contacts with health care personnel, and thereby more readily access to referrals to somatic units when needed. Hence, they might be diagnosed with various somatic conditions sooner or more frequently. Swedish national guidelines state that children and adolescents who undergo investigation according to structured protocols [for example for (ADHD) and major depressive disorder (MDD)] should also have a basic medical examination usually including routine laboratory tests, and individuals with suspected psychosis usually further have computed tomography (CT) scan or magnetic resonance imaging (MRI) and electroencephalography (EEG) carried out [28]. Accordingly, many Swedish departments of child and adolescent psychiatry request primary health care medical consultancy before a referral is accepted. Thus, many of the children who are diagnosed with psychiatric conditions are medically examined by a general practitioner or a pediatrician, and one can argue that the more an individual is examined the more likely that an illness will be found. However, the purpose of these examinations is to rule out medical causes for the symptoms expressed, and not to screen for illness in general. The detection of contingent somatic illness is still an expression for psychiatric-somatic comorbidity; however, one has to keep in mind that there is a possibility of positive bias in relation to the general child population.

Second, while the association between psychiatric and symptom-based diagnoses such as headache and myalgia could be impacted by diagnose making practices, the association between psychiatric disorders and metabolic or immunologic somatic disorders such as asthma, eczema, obesity and diabetes mellitus, raise questions about shared pathophysiological mechanisms. The role of the immune system in the development of psychiatric conditions such as depression has been discussed for decades. Increased inflammatory biomarkers such as C-reactive protein (CRP), interleukin 6 (IL-6) and tumor necrosis factor (TNF)-α have been found in patients with MDD [29], and some studies indicate normalization of inflammatory parameters along with treatment response [30]. Moreover, depressive like behavior has been experimentally induced in laboratory settings by administering inflammatory cytokines in both animals and humans [31]. Similarly, elevated levels of inflammatory cytokines have been found in patients with anxiety, schizophrenia and ADHD [32–34]. However, the studies on the latter diagnoses are sparser, and results more inconclusive. Likewise, the immune system plays a role in the development of several of the somatic conditions included in the present study. Given this background, shared immune-mediated pathophysiological processes do seem possible.

Third, recent research has focused on investigating the genetic overlap between somatic and mental disorders [35]. For example, Tylee and colleagues found significant genetic correlations between several different psychiatric and immune-related disorders [36]. However, correlations were weak or modest in general, and the clinical significance of these associations remains unknown. While single genetic polymorphisms are estimated to explain less than 0.1% of the phenotypic variance [37] genetic factors account for a considerable part of the etiology of most psychiatric disorders [38]. Moreover, environmental factors have been shown to directly influence genetic expression through epigenetic mechanisms, which could impact health and behavior later in life [39]. Considering the development and availability of advanced molecular methods, future studies could render new insights on comorbidity and the etiology of psychiatric disorders.

Finally, parental influence is an important factor in child and adolescent psychiatry, affecting environmental (as well as genetic) circumstances. Parents having children with ill-health might be more worried and/or prone to respond readily to symptoms and seek medical care. Dietary intake, physical activity levels, exposure to stressful life events and adherence to medical treatments and regimens are all factors of importance for health, and likewise under impact of parental influence during childhood. Moreover, parental neglect or other adverse childhood experience have been shown to impact mental as well as somatic health in adolescence [40, 41].

Indisputable, remarkably large differences in somatic illness between children with and without a psychiatric diagnose were found in the present study. Regardless of the direction of the association, or whether shared or separate etiologic pathways are at play, children and adolescents with mental illness are at great risk of concurrent somatic illness. This needs to be taken into account in health care planning as well as clinical settings to facilitate early detection and access to adequate treatment. Several areas in need of improvement for health care of adult psychiatric patients with somatic comorbidity have been addressed previously [1]. Integration of psychiatric and somatic health care needs to be optimized to facilitate service to this vulnerable and possibly health care-consuming group. Another obstacle is lack of consensus about treatment and prevention responsibility between health care units. The latter also includes funding issues. There is reason to believe that these impediments are just as valid in child and adolescent health care. Early detection of somatic illness or risk factors for chronic conditions could contribute to the implementation of interventions, and further on possible preventive measures in high risk groups. Thereby the risk for development of chronic conditions and complications could be reduced.

## Limitations

Although this is a large register-based study consisting of all 281,000 children and adolescents in the region of Västra Götaland, Sweden, with information on health care visits and diagnoses, there are a few important limitations that need to be discussed. First, only information on age, gender and diagnose was obtained for the study population. Socioeconomic factors are known to influence both somatic and mental health in children [42] and could act as a shared confounder. The Vega database holds no information on socioeconomic status. Second, the data collection was limited to a 3-year span, and no information on mental and/or somatic illness was obtained before and after the study period. However, it is plausible that this circumstance would impact both groups (with and without psychiatric diagnoses) equally. Third, the cross-sectional design does not allow for investigation of the direction of the relationship between psychiatric and somatic conditions. Longitudinal studies from childhood to adulthood would be of great value to further evaluate the development of comorbidity between psychiatric and somatic illness. Fourth, the present study made use of broad categories of psychiatric diagnoses based on the ICD 10 system. Another option would have been to use narrower diagnostic groups, for example neuropsychiatric diagnoses, or even separate diagnoses. Moreover, not all common childhood psychiatric conditions were analyzed (for example autism spectrum disorders). However, considering the use of register data and the known comorbidity between different psychiatric disorders during childhood, the intention was not to pinpoint associations between specific psychiatric and somatic disorders, but rather to examine a broader pattern of association. Additionally, to further address the association between mental and somatic health, use of self-reports (or parental reports) would be of great interest to catch not only the diagnosed cases, but also subclinical symptoms. In the present study, frequencies of somatic disorders were found to be somewhat lower than previously reported. This might be the result of using narrow diagnoses and only register data, that is, no clinical information. The Vega database covers all public and private health care in the region, and Västra Götaland region is representative of Sweden, indicating reliable data.

## Conclusions

The results of the present study show that children with psychiatric disorders are at remarkably high risk for concurrent somatic illness. The associations between psychiatric and somatic diagnoses span across all types of conditions investigated in the present study and across all ages. The direction of the relationship between psychiatric and somatic illness is not possible to demonstrate using cross-sectional data, and the investigation of causality was not the aim of the present study. However, the strength of the associations found, and the wide range of comorbidity shown, call for more research into the field. Yet, besides the scientifically interesting results, the study supports the importance of coordinated health care for children with psychiatric and somatic illness. Early detection of somatic illness or risk factors for chronic conditions could contribute to the implementation of interventions, and further on possible preventive measures in high-risk groups. Furthermore, increased knowledge and identification of comorbidity could reduce suffering in individual patients and result in a more effective use of resources in a population with high health care consumption.

## Electronic supplementary material

Below is the link to the electronic supplementary material.

Supplementary file1 (DOCX 32 kb)

## References

[1] Fleischhacker WW, Cetkovich-Bakmas M, De Hert M, Hennekens CH, Lambert M, Leucht S, Maj M, McIntyre RS, Naber D, Newcomer JW, Olfson M, Osby U, Sartorius N, Lieberman JA (2008). Comorbid somatic illnesses in patients with severe mental disorders: clinical, policy, and research challenges. J Clin Psychiatry.

[2] Westman J, Eriksson SV, Gissler M, Hällgren J, Prieto ML, Bobo WV, Frye MA, Erlinge D, Alfredsson L, Ösby U (2017). Increased cardiovascular mortality in people with schizophrenia: a 24-year national register study. Epidemiol Psychiatr Sci.

[3] Howard LM, Barley EA, Davies E, Rigg A, Lempp H, Rose D, Taylor D, Thornicroft G (2010). Cancer diagnosis in people with severe mental illness: practical and ethical issues. Lancet Oncol.

[4] Pillinger P, Beck K, Gobjila C, Donocik JG, Jauhar S, Howes OD (2017). Impaired glucose homeostasis in first-episode schizophrenia: a systematic review and meta-analysis. JAMA Psychiatry.

[5] Ross LE, Vigod S, Wishart J, Waese M, Spence JD, Oliver J, Chambers J, Anderson S, Shields R (2015). Barriers and facilitators to primary care for people with mental health and/or substance use issues: a qualitative study. BMC Fam Pract.

[6] Brämberg EB, Torgerson J, Kjellström AN, Welin P, Rusner M (2018). Access to primary and specialized health care for persons with severe mental illness: a qualitative study of perceived barriers and facilitators in Swedish health care. BMC Fam Pract.

[7] Ringen PA, Engh JA, Birkenaes I, Dieset I, Andreassen OA (2014). Increased mortality in schizophrenia due to cardiovascular disease—a non-systematic review of epidemiology, possible causes, and interventions. Front Psychiatry.

[8] Leucht S, Cipriani A, Spineli L, Mavridis D, Orey D, Richter F, Samara M, Barbui C, Engel RR, Geddes JR, Kissling W, Stapf MP, Lässig B, Salanti G, Davis JM (2015). Comparative efficacy and tolerability of 15 antipsychotic drugs in schizophrenia: a multiple-treatments meta-analysis. Lancet.

[9] Rapoport JL, Giedd JN, Gogtay N (2012). Neurodevelopmental model of schizophrenia: update 2012. Mol Psychiatry.

[10] Caspi A, Moffitt TE, Newman DL, Silva PA (1996). Behavioral observations at age 3 years predict adult psychiatric disorders: Longitudinal evidence from a birth cohort. Arch Gen Psychiatry.

[11] Pihlakoski L, Sourander A, Aromaa M, Rautava P, Helenius H, Sillanpaa M (2006). The continuity of psychopathology from early childhood to preadolescence: A prospective cohort study of 3–12-year-old children. Eur Child Adolesc Psychiatry.

[12] Spady DW, Schopflocher DP, Svenson LW, Thompson AH (2005). Medical and psychiatric comorbidity and health care use among Children 6–17 years old. Arch Pediatr Adolesc Med.

[13] Merikangas KR, Calkins ME, Burstein M, He JP, Chiavacci R, Lateef T, Ruparel K, Gur RC, Lehner T, Hakonarson H, Gur RE (2015). Comorbidity of physical and mental disorders in the neurodevelopmental genomics cohort study. Pediatrics.

[14] Muskens JB, Vermuelen K, Van Deurzen PAM, Tomesen EMA, Van der Gaag RJ, Buitelaar JK, Staal WG (2015). Somatische screening in kinder- en jeugd- psychiatrie: een descriptive pilotstudie. Tijdschr Psychatr.

[15] Sztein DM, Lane WG (2016). Examination of the comorbidity of mental illness and somatic conditions in hospitalized children in the United States Using the kids' inpatient database, 2009. Hosp Pediatr.

[16] Goodwin RD, Sourander A, Duarte CS, Niemela S, Multimäki K, Nikolakaros G, Helenius H, Piha J, Kumpulainen K, Moilanen I, Tamminen T, Almqvist F (2009). Do mental health problems in childhood predict chronic physical conditions among males in early adulthood? Evidence from a community-based prospective study. Psychol Med.

[17] Matheson BE, Eichen DM (2018). A review of childhood behavioral problems and disorders in the development of obesity: attention deficit/hyperactivity disorder, autism spectrum disorder, and beyond. Curr Obes Rep.

[18] Wilkes TCR, Guyn L, Li B, Lu M, Cawthorpe D (2012). Association of Child and Adolescent Psychiatric Disorders with somatic or biomedical diagnoses: do population-based utilization study results support the adverse childhood experiences study?. Perm J.

[19] Dudeney J, Sharpe L, Jaffe A, Jones EB, Hunt C (2017). Anxiety in youth with asthma: a meta-analysis. Pediatr Pulmonol.

[20] Brew BK, Lundholm C, Gong T, Larsson H, Almqvist C (2018). The familial aggregation of atopic diseases and depression or anxiety in children. Clin Exp Allergy.

[21] BeLue R, Francis LA, Colaco B (2009). Mental health problems and overweight in a nationally representative sample of adolescents: effects of race and ethnicity. Pediatrics.

[22] Buske-Kirschbaum A, Schmitt J, Plessow F, Romanos M, Weidinger S, Roessner V (2013). Psychoendocrine and psychoneuroimmunological mechanisms in the comorbidity of atopic eczema and attention deficit/hyperactivity disorder. Psychoneuroendocrinology.

[23] Yang CF, Yang CC, Wang IJ (2018). Association between allergic diseases, allergic sensitization and attention-deficit/hyperactivity disorder in children: a large-scale, population-based study. J Chin Med Assoc.

[24] Grey M, Whittemore R, Tamborlane W (2002). Depression in type 1 diabetes in children: natural history and correlates. J Psychosom Res.

[25] Khandaker GM, Zammit S, Lewis G, Jones PB (2014). A population-based study of atopic disorders and inflammatory markers in childhood before psychotic experiences in adolescence. Schizophr Res.

[26] Leonard BJ, Jang YP, Savik K, Plumbo PM, Christensen R (2002). Psychosocial factors associated with levels of metabolic control in youth with type 1 diabetes. J Pediatr Nurs.

[27] Cohen P, Pine DS, Must A, Kasen S, Brook J (1998). Prospective associations between somatic illness and mental illness from childhood to adulthood. Am J Epidemiol.

[28] Svenska Psykiatriska Föreningen (2009). Schizofreni: kliniska riktlinjer för utredning och behandling.

[29] Dowlati Y, Herrmann N, Swardfager W, Liu H, Sham L, Reim EK, Lanctôt KL (2010). A meta-analysis of cytokines in major depression. Biol Psychiatry.

[30] Schuebel K, Gitik M, Domschke K, Goldman D (2016). Making sense of epigenetics. Int J Neuropsychopharmacol.

[31] Miller AH, Maletic V, Raison CL (2009). Inflammation and its discontents: the role of cytokines in the pathophysiology of major depression. Biol Psychiatry.

[32] Haroon E, Raison CL, Miller AH (2012). Psychoneuroimmunology meets neuropsychopharmacology: translational implications of the impact of inflammation on behavior. Neuropsychopharmacology.

[33] Vogelzangs N, Beekman AT, de Jonge P, Penninx BW (2013). Anxiety disorders and inflammation in a large adult cohort. Transl Psychiatry.

[34] Müller N, Weidinger E, Leitner B, Schwarz MJ (2015). The role of inflammation in schizophrenia. Front Neurosci.

[35] Anand D, Colpo GD, Zeni G, Zeni CP, Teixeira AL (2017). Attention-deficit/hyperactivity disorder and inflammation: what does current knowledge tell us? A systematic review. Front Psychiatry.

[36] Bulik-Sullivan B, Finucane HK, Anttila V, Gusev A, Day FR, Loh PR, Duncan L, Perry JR, Patterson N, Robinson EB, Daly MJ, Price AL, Neale BM, ReproGen Consortium, Psychiatric Genomics Consortium, Genetic Consortium for Anorexia Nervosa of the Wellcome Trust Case Control Consortium 3 (2015). An atlas of genetic correlations across human diseases and traits. Nat Genet.

[37] Tylee DS, Sun J, Hess JL, Tahir MA, Sharma E, Malik R, Worrall BB, Levine AJ, Martinson JJ, Nejentsev S, Speed D, Fischer A, Mick E, Walker BR, Crawford A, Grant SFA, Polychronakos C, Bradfield JP, Sleiman PMA, Hakonarson H, Ellinghaus E, Elder JT, Tsoi LC, Trembath RC, Barker JN, Franke A, Dehghan A, Faraone SV, Glatt SJ, 23 and Me Research Team, Inflammation Working Group of the CHARGE Consortium, METASTROKE Consortium of the International Stroke Genetics Consortium, Netherlands Twin Registry, neuroCHARGE Working Group, Obsessive Compulsive and Tourette Syndrome Working Group of the Psychiatric Genomics Consortium (2018). Genetic correlations among psychiatric and immune-related phenotypes based on genome-wide association data. Am J Med Genet B Neuropsychiatr Genet.

[38] Munafo MR, Zammit S, Flint J (2014). Practitioner review: A critical perspective on gene-environment interaction models - what impact should they have on clinical perceptions and practice?. J Child Psychol Psychiatry Allied Discip.

[39] Kendler KS (2013). What psychiatric genetics has taught us about the nature of psychiatric illness and what is left to learn. Mol Psychiatry.

[40] Silverman AB, Reinherz HZ, Gianconia RM (1996). The long-term sequelae of child and adolescent abuse: a longitudinal community study. Child Abuse Negl.

[41] Flaherty EG, Thompson R, Dubowitz H, Harvey EM, English DJ, Proctor LJ, Runyan DK (2013). Adverse childhood experiences and child health in early adolescence. JAMA Pediatr.

[42] Reiss F (2013). Socioeconomic inequalities and mental health problems in children and adolescents: a systematic review. Soc Sci Med.

