# Supplementary material for: Somatic comorbidity in children and adolescents with psychiatric disorders
Source: Eur Child Adolesc Psychiatry. 2019 Mar 20;28(11):1517–25. doi: 10.1007/s00787-019-01313-9 (PMC6800882; doi:10.1007/s00787-019-01313-9)
Supplement: Supplementary file 1 — Supplementary file1 (DOCX 32 kb) [file 787_2019_1313_MOESM1_ESM.docx]

Article title: Somatic comorbidity in children and adolescents with psychiatric disorders

Journal name: European Child and Adolescent Psychiatry

Author names: Sara Agnafors, Anna Norman Kjellström, Jarl Torgerson, Marie Rusner

Corresponding author: Sara Agnafors, Division of Children’s and Women’s health, Institution of Clinical and Experimental Medicine, Linköping University, Sweden

Email: sara.agnafors@liu.se

**Supplementary 1. Group differences in somatic diagnoses between children with anxiety diagnoses and those without during the study period 2011-2013. Crosstabs with Chi square.**

**Age 3-5**

| Somatic diagnose | Anxiety  No (n=56752) | Yes (n=89) |
| --- | --- | --- |
| Obesity | X |  |
| Asthma* | 6.9 % (n=3942) | 12.4 % (n=11) |
| Diabetes type 1 | NS |  |
| Bowel | 5.6 % (n=3161) | 20.2 % (18) |
| Eczema | 7.7 % (n=4374) | 23.6 % (n=21) |
| Myalgia | NS |  |
| Migraine | NS |  |
| Headache | NS |  |

**Age 6-8**

| Somatic diagnose | Anxiety  No (n=53024) | Yes (n=355) |
| --- | --- | --- |
| Obesity* | 1.2 % (n=616) | 3.1 % (n=11) |
| Asthma | 5.2 % (n=2749) | 15.5 % (n=55) |
| Diabetes type 1 | NS |  |
| Bowel | 4.3 % (n=2278) | 14.6 % (n=52) |
| Eczema | 6.3 % (n=3353) | 12.4 % (n=44) |
| Myalgia | 0.9 % (n=475) | 4.8 % (n=17) |
| Migraine | 0.4 % (n=230) | 2.0 % (n=7) |
| Headache | X |  |

**Age 9-11**

| Somatic diagnose | Anxiety  No (n=49383) | Yes (n=928) |
| --- | --- | --- |
| Obesity | 1.7 % (n=847) | 4.4 % (n=41) |
| Asthma | 5.0 % (n=2451 | 14.2 % (n=132) |
| Diabetes typ 1* | 0.4 % (n=209) | 1.0 % (n=9) |
| Bowel | 3.4 % (n=1701) | 14.1 % (n=131) |
| Eczema | 5.5 % (n=2751) | 13.3 % (n=123) |
| Myalgia | 1.5 % (n=755) | 6.3 % (n=58) |
| Migraine | 0.7 % (n=331) | 3.1 % (n=29) |
| Headache | 0.5 % (n=261) | 2.0 % (n=19) |

**Age 12-14**

| Somatic diagnose | Anxiety  No (n=47157) | Yes (n=1432) |
| --- | --- | --- |
| Obesity | 1.6 % (n=754) | 4.5 % (n=64) |
| Asthma | 5.1 % (n=2401) | 12.3 % (n=176) |
| Diabetes type 1* | 0.6 % (n=293) | 1.2 % (n=17) |
| Bowel | 2.2 % (n=1047) | 10.1 % (n=145) |
| Eczema | 4.5 % (n=2131) | 10.0 % (n=143) |
| Myalgia | 2.1 % (n=968) | 6.6 % (n=94) |
| Migraine | 1.1 % (n=516) | 3.4 % (n=48) |
| Headache | 0.8 % (n=370) | 3.9 % (n=56) |

**Age 15-18**

| Somatic diagnose | Anxiety  No (n=68796) | Yes (n=3560) |
| --- | --- | --- |
| Obesity | 1.0 % (n=665) | 3.6 % (n=128) |
| Asthma | 3.8 % (n=2626) | 10.3 % (n=366) |
| Diabetes type 1* | 0.7 % (n=487) | 1.2 % (n=43) |
| Bowel | 1.8 % (n=1213) | 8.6 % (n=305) |
| Eczema | 4.3 % (n=2961) | 12.3 % (n=437) |
| Myalgia | 2.6 % (n=1759) | 8.5 % (n=304) |
| Migraine | 1.0 % (n=666) | 4.2 % (n=148) |
| Headache | 1.1 % (n=739) | 5.3 % (n=190) |

**Age 3-18**

| Somatic diagnose | Anxiety  No (n=275112) | Yes (n=6364) |
| --- | --- | --- |
| Obesity | 1.1 % (n=3146) | 3.9 % (n=247) |
| Asthma | 5.2 % (n=14169) | 11.6 % (n=740) |
| Diabetes type 1 | 0.4 % (n=1219) | 1.1 % (n=72) |
| Bowel | 3.4 % (n=9400) | 10.2 % (n=651) |
| Eczema | 5.6 % (n=15534) | 12.1 % (n=768) |
| Myalgia | 1.5 % (n=4214 | 7.4 % (n=473) |
| Migraine | 0.7 % (n=1825) | 3.6 % (n=232) |
| Headache | 0.6 % (n=1514) | 4.2 % (n=270 |

Note: X = 5 individuals or fewer. p<0.001, p<0.05 indicated with *.

**Supplementary 2. Group differences in somatic diagnoses between children with affective diagnoses and those without during the study period 2011-2013. Crosstabs with Chi square.**

**Age 3-5**

| Somatic diagnose | Affective  No (n=56833) | Yes (n=8) |
| --- | --- | --- |
| Obesity | NS |  |
| Asthma | NS |  |
| Diabetes type 1 | NS |  |
| Bowel | NS |  |
| Eczema | NS |  |
| Myalgia | NS |  |
| Migraine | NS |  |
| Headache | NS |  |

**Age 6-8**

| Somatic diagnose | Affective  No (n=53321) | Yes (n=58) |
| --- | --- | --- |
| Obesity | NS |  |
| Asthma* | 5.2 % (n=2796) | 13.8 % (n=8) |
| Diabetes type 1 | X |  |
| Bowel | NS |  |
| Eczema* | 6.4 % (n=3389) | 13.8 % (n=8) |
| Myalgia | X |  |
| Migraine | NS |  |
| Headache | NS |  |

**Age 9-11**

| Somatic diagnose | Affective  No (n=50096) | Yes (n=215) |
| --- | --- | --- |
| Obesity | NS |  |
| Asthma* | 5.1 % (n=2563) | 9.3 % (n=20) |
| Diabetes type 1 | NS |  |
| Bowel | 3.6 % (n=1810) | 10.2 % (n=22) |
| Eczema | 5.6 % (n=2809) | 13.5 % (n=29) |
| Myalgia* | 1.6 % (n=804) | 4.2 % (n=9) |
| Migraine | X |  |
| Headache | 0.5 % (n=274) | 2.8 % (n=6) |

**Age 12-14**

| Somatic diagnose | Affective  No (n=47935) | Yes (n=38) |
| --- | --- | --- |
| Obesity | 1.6 % (n=780) | 5.8 % (n=38) |
| Asthma | 5.2 % (n=2512) | 9.9 % (n=65) |
| Diabetes type 1 | NS |  |
| Bowel | 2.4 % (n=1136) | 8.6 % (n=56) |
| Eczema | 4.6 % (n=2207) | 10.2 % (n=67) |
| Myalgia | 2.1 % (n=1017) | 6.9 % (n=45) |
| Migraine | 1.1 % (n=541) | 3.5 % (n=23) |
| Headache | 0.8 % (n=391) | 5.4 % (n=35) |

**Age 15-18**

| Somatic diagnose | Affective  No (n=70083) | Yes (n=2273) |
| --- | --- | --- |
| Obesity | 1.0 % (n=699) | 4.1 % (n=94) |
| Asthma | 4.0 % (n=2779) | 9.4 % (n=213) |
| Diabetes type 1* | 0.7 % (n=505) | 1.1 % (n=25) |
| Bowel | 1.9 % (n=1327) | 8.4 % (n=191) |
| Eczema | 4.4 % (n=3113) | 12.5 % (n=285) |
| Myalgia | 2.7 % (n=1885) | 7.8 % (n=178) |
| Migraine | 1.0 % (n=716) | 4.3 % (n=98) |
| Headache | 1.2 % (n=818) | 4.9 % (n=111) |

**Age 3-18**

| Somatic diagnose | Affective  No (n=278268) | Yes (n=3208) |
| --- | --- | --- |
| Obesity | 1.2 % (n=3252) | 4.4 % (n=141) |
| Asthma | 5.2 % (n=14602) | 9.6 % (n=307) |
| Diabetes type 1 | 0.5 % (n=1254) | 1.2 % (n=37) |
| Bowel | 3.5 % (n=9777) | 8.5 % (n=274) |
| Eczema | 5.7 % (n=15912) | 12.2 % (n=390) |
| Myalgia | 1.6 % (n=4453) | 7.3 % (n=234) |
| Migraine | 0.7 % (n=1932) | 3.9 % (n=125) |
| Headache | 0.6 % (n=1659) | 4.7 % (n=152) |

Note: X= 5 individuals or fewer. p<0.001, p<0.05 indicated with *.

**Supplementary 3. Group differences in somatic diagnoses between children with behavioral diagnoses and those without during the study period 2011-2013. Crosstabs with Chi square.**

**Age 3-5**

| Somatic diagnose | Behavior  No (n=55851) | Yes (n=990) |
| --- | --- | --- |
| Obesity | 0.4 % (n=249) | 1.8 % (n=18) |
| Asthma | 6.7 % (n=3767) | 18.8 % (n=186) |
| Diabetes type 1 | X |  |
| Bowel | 5.3 % (n=2950) | 23.1 % (n=229) |
| Eczema | 7.5 % (n=4184) | 21.3 % (n=211) |
| Myalgia | 0.4 % (n=245) | 1.2 % (n=12) |
| Migraine | X |  |
| Headache | X |  |

**Age 6-8**

| Somatic diagnose | Behavior  No (n=51070) | Yes (n=2309) |
| --- | --- | --- |
| Obesity | 1.1 % (n=544) | 3.6 % (n=83) |
| Asthma | 4.9 % (n=2493) | 13.5 % (n=311) |
| Diabetes type 1 | 0.3 % (n=128 | 0.7 % (n=16) |
| Bowel | 3.8 % (n=1933) | 17.2 % (n=397) |
| Eczema | 6.0 % (n=3057) | 14.7 % (n=340) |
| Myalgia | 0.9 % (n=435) | 2.5 % (n=57) |
| Migraine | 0.4 % (n=207) | 1.3 % (n=30) |
| Headache | 0.2 % (n=127) | 0.7 % (n=16) |

**Age 9-11**

| Somatic diagnose | Behavior  No (n=48170) | Yes (n=2141) |
| --- | --- | --- |
| Obesity | 1.6 % (n=756) | 6.2 % (n=132) |
| Asthma | 4.8 % (n=2332) | 11.7 % (n=251) |
| Diabetes type 1 | 0.4 % (n=197) | 1.0 % (n=21) |
| Bowel | 3.3 % (n=1574) | 12.1 % (n=258) |
| Eczema | 5.4 % (n=2592) | 11.5 % (n=246) |
| Myalgia | 1.5 % (n=746) | 3.1 % (n=67) |
| Migraine | 0.7 % (n=329) | 1.4 % (n=31) |
| Headache | 0.5 % (n=250) | 1.4 % (n=30) |

**Age 12-14**

| Somatic diagnose | Behavior  No (n=46639) | Yes (n=1950) |
| --- | --- | --- |
| Obesity | 1.5 % (n=702) | 5.9 % (n=116) |
| Asthma | 5.1 % (n=2376) | 10.3 % (n=201) |
| Diabetes type 1* | 0.6 % (n=288) | 1.1 % (n=22) |
| Bowel | 2.3 % (n=1065) | 6.5 % (n=127) |
| Eczema | 4.5 % (n=2116) | 8.1 % (n=158) |
| Myalgia | 2.1 % (n=983) | 4.1 % (n=79) |
| Migraine | 1.1 % (n=523) | 2.1 % (n=41) |
| Headache* | 0.9 % (n=400) | 1.3 % (n=26) |

**Age 15-18**

| Somatic diagnose | Behavior  No (n=70017) | Yes (n=2339) |
| --- | --- | --- |
| Obesity | 1.0 % (n=670) | 5.5 % (n=123) |
| Asthma | 4.0 % (n=2768) | 9.6 % (n=224) |
| Diabetes type 1 | 0.7 % (n=494) | 1.5 % (n=36) |
| Bowel | 2.0 % (n=1374) | 6.2 % (n=144) |
| Eczema | 4.5 % (n=3169) | 9.8 % (n=229) |
| Myalgia | 2.8 % (n=1937) | 5.4 % (n=126) |
| Migraine | 1.1 % (n=748) | 2.8 % (n=66) |
| Headache | 1.2 % (n=874) | 2.4 % (n=55) |

**Age 3-18**

| Somatic diagnose | Behavior  No (n=271747) | Yes (n=9729) |
| --- | --- | --- |
| Obesity | 1.1 % (n=2921) | 4.9 % (n=472) |
| Asthma | 5.1 % (n=13736) | 12.1 % (n=1173) |
| Diabetes type 1 | 0.4 % (n=1192) | 1.0 % (n=99) |
| Bowel | 3.3 % (n=8896) | 11.9 % (n=1155) |
| Eczema | 5.6 % (n=15118) | 12.2 % (n=1184) |
| Myalgia | 1.6 % (n=4346) | 3.5 % (n=341) |
| Migraine | 0.7 % (n=1885) | 1.8 % (n=172) |
| Headache | 0.6 % (n=1680) | 1.3 % (n=131) |

Note: X = 5 individuals or fewer. p<0.001, p<0.05 indicated with *.

**Supplementary 4. Group differences in somatic diagnoses between children with diagnoses of psychotic conditions and those without during the study period 2011-2013. Crosstabs with Chi square.**

**Age 12-17**

| Somatic diagnose | Psychotic conditions  No (n=120869) | Yes (n=76) |
| --- | --- | --- |
| Obesity | X |  |
| Asthma* | 4.6 % (n=5560) | 11.8 % (n=9) |
| Diabetes type 1 | X |  |
| Bowel* | 2.2 % (n=2704) | 7.9 % (n=6) |
| Eczema | NS |  |
| Myalgia | 2.6 % (n=3118) | 9.2 % (n=7) |
| Migraine | X |  |
| Headache | X |  |

Note: X = 5 individuals or fewer. Statistical significance set to p<0.001, p<0.05 indicated with *.

**Supplementary 5. Group differences in somatic diagnoses between children with diagnoses of substance use and those without during the study period 2011-2013. Crosstabs with Chi square.**

**Age 9-17**

| Somatic diagnose | Substance use  No (n=170292) | Yes (n=964) |
| --- | --- | --- |
| Obesity | 1.5 % (n=2472) | 2.8 % (n=27) |
| Asthma | 4.7 % (n=8038) | 11.8 % (n=114) |
| Diabetes type 1 | 0.6 % (n=1039) | 2.0 % (n=19) |
| Bowel | 2.6 % (n=4482) | 6.2 % (n=60) |
| Eczema | 4.9 % (n=8403) | 11.1 % (n=107) |
| Myalgia | 2.3 % (n=3869) | 7.2 % (n=69) |
| Migraine | 1.0 % (n=1705) | 3.4 % (n=33) |
| Headache | 0.9 % (n= 1595) | 4.1 % (n=40) |

Note: All analyses p <0.001.
